# Endoscopic submucosal dissection of recurrent duodenal adenoma: combined use of multiple strategies for a difficult case

**DOI:** 10.1055/a-2239-3182

**Published:** 2024-02-07

**Authors:** Ludovico Alfarone, Jérémie Albouys, Romain Legros, Mathieu Pioche, Timothée Wallenhorst, Sophie Geyl, Jérémie Jacques

**Affiliations:** 1 Gastroenterology and Endoscopy Unit, Dupuytren University Hospital, Limoges, France; 2 IRCCS Humanitas Research Hospital, Rozzano, Milan, Italy; 3 Gastroenterology and Endoscopy Unit, Edouard Herriot Hospital, Lyon, France; 4 Endoscopy and Gastroenterology Unit, Pontchaillou University Hospital, Rennes, France

The duodenal anatomy, which involves a thin muscle layer and rich vascularization, makes endoscopic resection harder and more dangerous in this region than in other regions of the gastrointestinal tract. Additionally, the physiological shape of the stomach decreases endoscope maneuverability. Thus, large non-ampullary duodenal adenomas are usually removed through snare-based techniques because endoscopic submucosal dissection (ESD) is considered excessively hazardous. However, in select difficult cases, ESD may be the sole viable option for successful resection 1 .

A 73-year-old woman was referred to our unit for resection of a Paris IIa 25-mm non-ampullary duodenal adenoma near the major papilla. The adenoma had been treated by surgical mucosectomy more than 10 years prior to referral. After 30 min of attempting to remove it by piecemeal endoscopic mucosal resection, which was unsuccessful because the lesion could not be retrieved with a snare, we converted the procedure to ESD ( Video 1 ).

Underwater traction-assisted endoscopic submucosal dissection of a recurrent duodenal adenoma using the pocket-creation method.Video 1

Because the scope maneuverability was poor, we used the pocket-creation method, beginning with incision and trimming on the oral side. We then placed a countertraction system consisting of two clips and a rubber band. The use of traction combined with the saline-immersion technique provided complete exposure of the dissection plane ( Fig. 1 ), which was immediately below the hard fibrotic area; accurate dissection was then performed 2 3 ( Fig. 2 ). The use of a scissor-type knife was required to complete the dissection because the anal side of the lesion could not be easily accessed. Finally, we used a side-viewing scope to close the scar, exercising caution to avoid grasping the major papilla ( Fig. 3 ). En bloc R0 resection of the adenoma, which exhibited high-grade dysplasia, was achieved. No adverse events occurred.

**Fig. 1:**
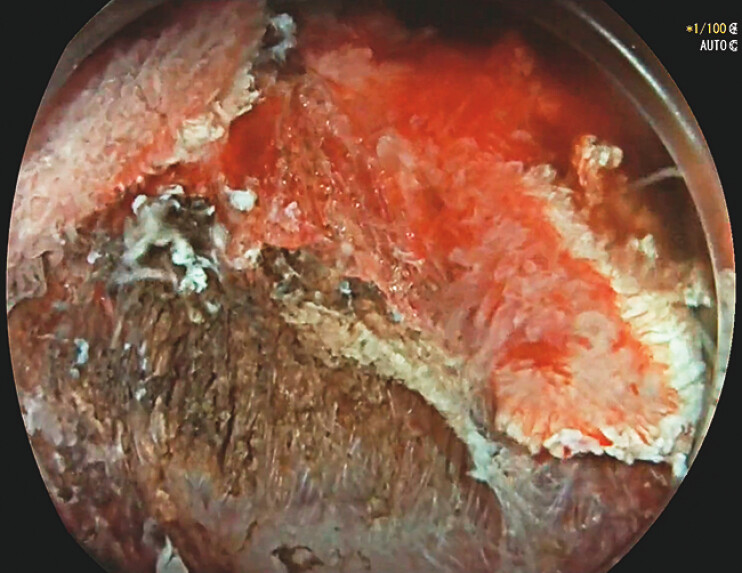
Optimal exposure of submucosal space.

**Fig. 2:**
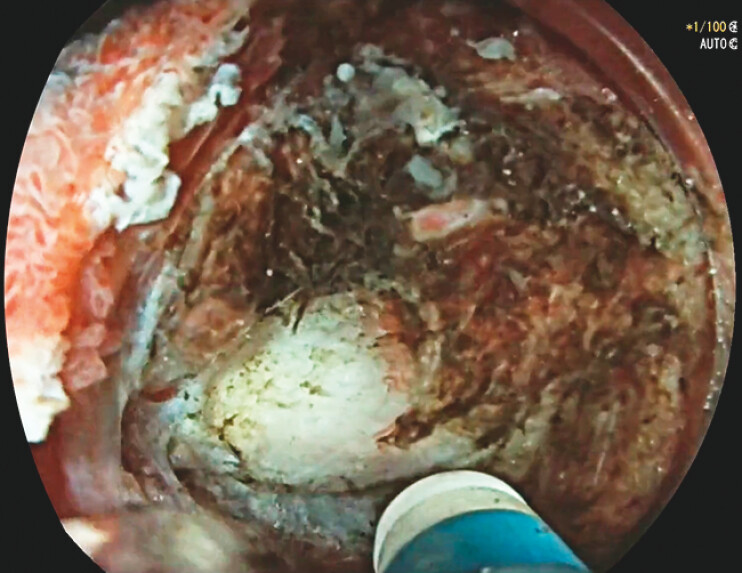
Dissection under the fibrosis.

**Fig. 3:**
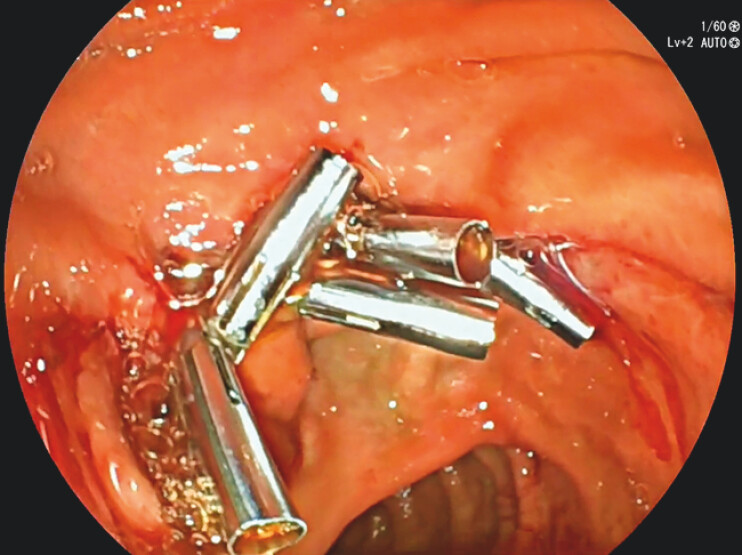
Complete closure of scar, sparing the major papilla.

Indications for duodenal ESD are rare. Combinations of complementary strategies such as the pocket-creation method, saline-immersion technique, and traction are essential in these challenging cases.

Endoscopy_UCTN_Code_TTT_1AO_2AG

**Correction**

**Correction: Endoscopic submucosal dissection of recurrent duodenal adenoma: combined use of multiple strategies for a difficult case** Alfarone Ludovico, Albouys Jérémie, Legros Romain et al. Correction: Endoscopic submucosal dissection of recurrent duodenal adenoma: combined use of multiple strategies for a difficult case. Endoscopy 2024; 56: E595–E597. doi:10.1055/a-2239-3182 In the above-mentioned article affiliation 2 has been corrected. Correct is: IRCCS Humanitas Research Hospital, Rozzano, Milan, Italy. This was corrected in the online version on July 31, 2025.

## Notes

Endoscopy E-Videos https://eref.thieme.de/e-videos
*E-Videos* is an open access online section of the journal *Endoscopy* , reporting on interesting cases and new techniques in gastroenterological endoscopy. All papers include a high-quality video and are published with a Creative Commons CC-BY license. Endoscopy E-Videos qualify for HINARI discounts and waivers and eligibility is automatically checked during the submission process. We grant 100% waivers to articles whose corresponding authors are based in Group A countries and 50% waivers to those who are based in Group B countries as classified by Research4Life (see: https://www.research4life.org/access/eligibility/ ). This section has its own submission website at https://mc.manuscriptcentral.com/e-videos .

## Notes

Correction **Correction: Endoscopic submucosal dissection of recurrent duodenal adenoma: combined use of multiple strategies for a difficult case** Alfarone Ludovico, Albouys Jérémie, Legros Romain et al. Correction: Endoscopic submucosal dissection of recurrent duodenal adenoma: combined use of multiple strategies for a difficult case. Endoscopy 2024; 56: E595–E597. doi:10.1055/a-2239-3182 In the above-mentioned article affiliation 2 has been corrected. Correct is: IRCCS Humanitas Research Hospital, Rozzano, Milan, Italy. This was corrected in the online version on July 31, 2025.
