# Correction: Endoscopic submucosal dissection of recurrent duodenal adenoma: combined use of multiple strategies for a difficult case

**DOI:** 10.1055/a-2664-6227

**Published:** 2025-07-31

**Authors:** Ludovico Alfarone, Jérémie Albouys, Romain Legros, Mathieu Pioche, Timothée Wallenhorst, Sophie Geyl, Jérémie Jacques

**Affiliations:** 1 Gastroenterology and Endoscopy Unit, Dupuytren University Hospital, Limoges, France; 2 IRCCS Humanitas Research Hospital, Rozzano, Milan, Italy; 3 Gastroenterology and Endoscopy Unit, Edouard Herriot Hospital, Lyon, France; 4 Endoscopy and Gastroenterology Unit, Pontchaillou University Hospital, Rennes, France



In the above-mentioned article affiliation 2 has been corrected. Correct is: IRCCS Humanitas Research Hospital, Rozzano, Milan, Italy. This was corrected in the online version on July 31, 2025.

